# Engraftment of wild-type alveolar type II epithelial cells in surfactant protein C deficient mice

**DOI:** 10.21203/rs.3.rs-4673915/v1

**Published:** 2024-09-13

**Authors:** Camilla Predella, Lauren Lapsley, Keyue Ni, John W. Murray, Hsiao-Yun Liu, Joshua E. Motelow, Hans-Willem Snoeck, Stephan W. Glasser, Anjali Saqi, N. Valerio Dorrello

**Affiliations:** 1 Division of Pediatric Critical Care Medicine and Hospital Medicine, Department of Pediatrics, Columbia University Vagelos College of Physicians and Surgeons, New York, NY, USA; 2 Department of Chemistry, Materials and Chemical Engineering “G. Natta”, Politecnico of Milan, Milan, Italy; 3 Department of Electronics, Information and Bioengineering, Politecnico of Milan, Milan, Italy; 4 Columbia Center for Human Development, Columbia University Vagelos College of Physicians and Surgeons, New York, NY, USA; 5 Department of Medicine, Columbia University Irving Medical Center, New York, NY, USA; 6 Department of Pathology and Cell Biology, Columbia University Vagelos College of Physicians and Surgeons and New York-Presbyterian, New York, NY, USA; 7 Medical Sciences Program, Department of Medical Education, University of Cincinnati College of Medicine, Cincinnati, Ohio, USA; 8 Division of Pediatric Critical Care Medicine and Hospital Medicine, Department of Pediatrics, Columbia University Vagelos College of Physicians and Surgeons and New York-Presbyterian Morgan Stanley Children’s Hospital, New York, NY, USA

**Keywords:** cell therapy, surfactant deficiency, childhood interstitial lung disease, alveolar type II epithelial cell, bleomycin

## Abstract

Childhood interstitial lung disease (chILD) secondary to pulmonary surfactant deficiency is a devastating chronic lung disease in children. Clinical presentation includes mild to severe respiratory failure and fibrosis. There is no specific treatment, except lung transplantation, which is hampered by a severe shortage of donor organs, especially for young patients. Repair of lungs with chILD represents a longstanding therapeutic challenge but cell therapy is a promising strategy. As surfactant is produced by alveolar type II epithelial (ATII) cells, engraftment with normal or gene-corrected ATII cells might provide an avenue to cure. Here we used a chILD disease-like model, *Sftpc*^−*/*−^ mice, to provide proof-of-principle for this approach. *Sftpc*^−*/*−^ mice developed chronic interstitial lung disease with age and were hypersensitive to bleomycin. We could engraft wild-type ATII cells after low dose bleomycin conditioning. Transplanted ATII cells produced mature SPC and attenuated bleomycin-induced lung injury up to two months post-transplant. This study demonstrates that partial replacement of mutant ATII cells can promote lung repair in a mouse model of chILD-like disease.

## Introduction

Pulmonary surfactant lowers surface tension in the alveoli, thus preventing alveolar expiratory collapse and reducing the work of breathing. It is produced and recycled in lamellar bodies (LBs) of alveolar epithelial type II (ATII) cells starting at 24 weeks gestational age^1^. Surfactant is composed of ~90% lipids and 8–10% surfactant proteins (SP) A, B, C, and D^2^. 50–85% of surfactant is recycled by ATII cells while the remainder is degraded by alveolar macrophages^2–5^. Children with pathogenic DNA sequence variants disrupting normal pulmonary surfactant production develop childhood interstitial lung disease (chILD)^6–11^. ChILD secondary to surfactant defects is caused by mutations in: *(i) SFTPB* (MIM: 178640), and *SFTPC* (MIM: 610913), which encode surfactant protein components; *(ii) ABCA3* (MIM: 610921), which encodes ATP-binding cassette sub-family A member 3, localized on the limiting membrane of LBs and essential for secretion; and *(iii) NKX2.1* (MIM: 610978), which encodes a transcription factor regulating *SFTPB*, *SFTPC* and *ABCA3*^6,7,9^.

The incidence of chILD ranges from 0.1 to 16.2 per 100,000 people^12,13^ with a prevalence of 1.3–3.8 per million and mortality as high as 35%^6,9–11,14–16^. Some forms of chILD are lethal in the neonatal period while others cause respiratory disease ranging from neonatal respiratory failure to childhood- or adult-onset interstitial lung disease. The pathology of chILD includes ATII hyperplasia, interstitial thickening, foamy macrophages, alveolar proteinosis, and interstitial pneumonitis^7,10^. This pathology is explained at least in part by the fact that defective ATII cells affect alveolar homeostasis and cause fibrotic lung remodeling, as ATII cells are facultative progenitors of the alveolar epithelium after injury^17–24^. In mice, for example, partial depletion of ATII cells increased susceptibility to bleomycin-induced fibrosis while total depletion of ATII cells caused extensive spontaneous lung fibrosis^25–27^.

Supportive therapy, such as corticosteroids, hydroxychloroquine, and azithromycin has limited benefit for the majority of cases leaving lung transplantation as the *only* definitive treatment^6,9,28^. Lung transplantation remains limited by the shortage of suitable donor organs, especially for smaller children^29,30^. Moreover, the overall survival rate at five years post-transplant is just 55%, a figure that has not significantly improved in the last 20 years^30^. There is therefore an urgent need for improved treatments.

Cell therapy, in which gene-corrected cells are transplanted into the lungs to replace defective ATII cells, is an exciting treatment option for chILD. In rats, intratracheal (i.t.) administration of ATII cells after bleomycin injury may result in reduction of fibrosis^31,32^. In mice infected with H1N1 influenza, oxygen saturation improved after ATII cell transplant^33^. It is unknown whether transplanted ATII cells could affect the disease, and if so, how many endogenous cells need to be replaced with exogenous ATII cells for therapeutic effect.

We investigated the efficacy of cell therapy in a viable mouse model of chILD-like disease, the *Sftpc*^−*/*−^ mouse. In humans, *SFTPC* encodes a 191 or 197 amino acid protein (length varies due to alternative splicing) that is palmitoylated, proteolytically processed in the endoplasmic reticulum, and shuttled to the multivesicular bodies where final cleavage generating mature SPC (35 amino acids) takes place. SPC is then incorporated into LBs in which surfactant is stored prior to secretion in the alveolar space^2,34^. Clinically, mutations in *SFTPC* have variable penetrance with phenotypes ranging from neonatal respiratory failure to chILD to idiopathic pulmonary fibrosis in adulthood. Some patients require lung transplant, others are managed with long-term mechanical ventilation, and others remain almost asymptomatic. Postnatal mortality is rare^35,36^. The most common human *SFTPC* mutation to cause chILD is the threonine-to-isoleucine substitution at the codon 73 (*SFTPC*^*I73T*^) resulting in mis-trafficking of SPC with proteotoxicity^37^. One study has demonstrated successful *in utero* gene editing using a mouse model of the most common *SFPTC* mutation responsible for chILD, *Sftpc*^*I73T*^.^38^ To be clinically applicable, diagnosis and correction have to occur before birth, which is not yet feasible^38^.

While our animal model is a double knockout which is not a genetic mimic of the most common pathogenic human *SFTPC* mutation, it recapitulates three critical characteristics of chILD: lack of normal surfactant, fibrosis, progressive lung disease with age.

In this proof-of-concept study, we used *Sftpc*^−*/*−^ mice to show, for the first time, an effective cell therapy in an animal model of chILD-like disease.

## Results

### Morphological changes of the lung in Sftpc^−/−^ mice.

**Figure 1.**
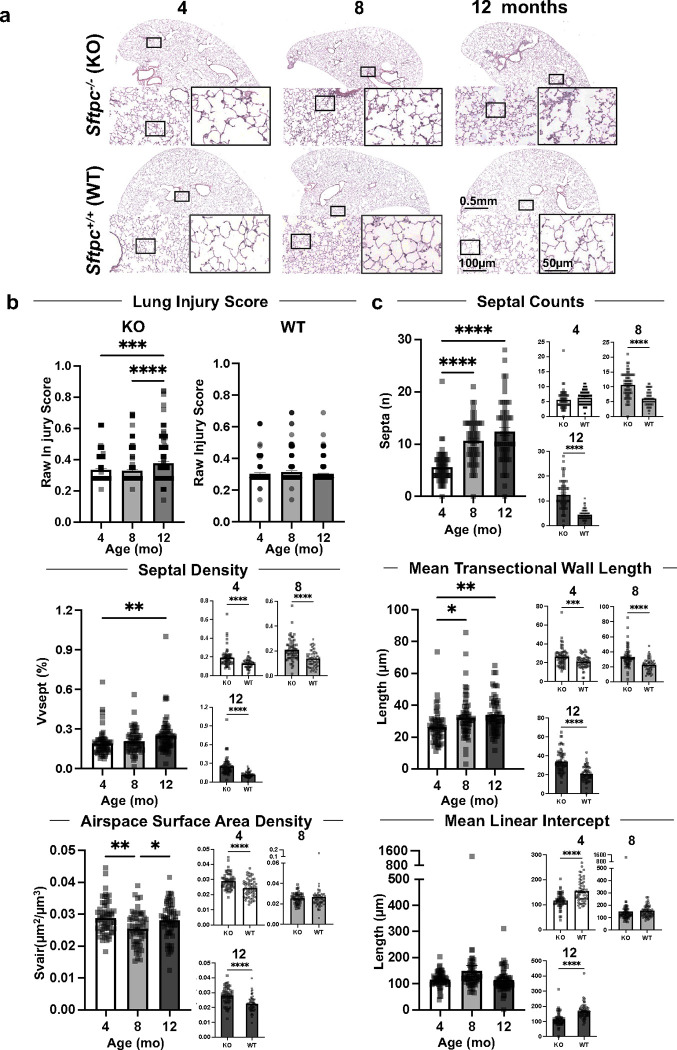
Morphological and stereological lung analysis of *Sftpc*^*−/−*^ mice at different ages. **a.** Representative histologic sections of whole lung comparing *Sftpc*^*−/−*^ and *Sftpc*^*+/+*^ mice at 4, 8, 12 months old. Black squares indicate the area shown in the higher magnification. **b-c.** LIS, morphometric, and stereological lung analysis are evaluated on 1000×1000 μm lung histology fields (n=60 ROI/mouse, 3 mice/group). Data are reported as mean± SEM and analyzed by one-way ANOVA when the comparison is within different ages of the same strain and by Student t-test when KO is compered to WT: LIS (***, p=0.0006; ****, p<0.0001). Septal counts (****,p<0.0001). Septal density (**, p=0.0090; ****, p<0.0001). Mean trans-sectional wall length (*, p=0.0115; **, p=0.0011; ***, p=0.009; ****, p<0.0001). Mean linear intercept (****, p<0.0001) and airspace surface area density (*, p=0.0291; **, p=0.0025; ****, p<0.0001).

**Figure 2.**
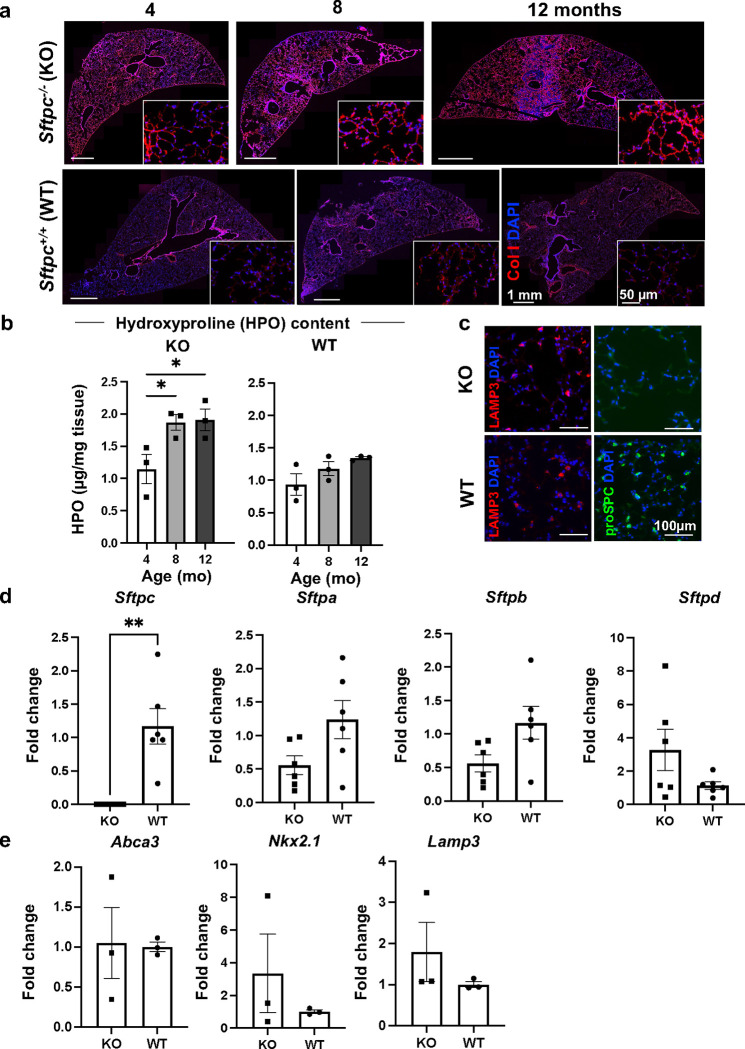
Lung collagen content and ATII cell markers analysis in *Sftpc*^*−/−*^ mice at different ages. **a.** Collagen I immunostaining of representative lung sections of 4, 8, 12 months old comparing *Sftpc*^*−/−*^ and *Sftpc*^*+/+*^ mice. **b.** Hydroxyproline content quantification via colorimetric hydroxyproline. Data are reported as mean±SEM, n=3, and analyzed by one-way ANOVA (*, p=0.0398–0.0495). **c.** Representative immunofluorescent staining of ATII cells in *Sftpc*^*−/−*^ (Lamp3^+^/proSPC^−^) and *Sftpc*^*+/+*^ (Lamp3^+^/proSPC^+^) mice. **d-e.** Expression by RT-qPCR of mRNA for surfactant proteins **(d)** and alveolar epithelial markers **(e).** Data are reported as mean±SEM, n=3–6, and analyzed by one-way ANOVA (**, p=0.0013).

Previous work showed that *Sftpc*^−*/*−^ mice on a 129/Sv background developed an interstitial pneumonitis-like phenotype beginning at two months of age and worsening with age up to 12–14 months^39^. The phenotype was characterized by irregular alveolar septation, mononucleated cell infiltrates, ATII cell hyperplasia, and interstitial thickening^39^. To develop a time course of the disease, we analyzed the lung histology and stereology in *Sftpc*^−*/*−^ (knock out, KO) and *Sftpc*^*+/+*^ (wild-type, WT) mice at 4-, 8-, and 12-month old (mo) (Figure 1). Histological analysis of lung sections showed progressive lung injury characterized by neutrophil infiltration into interstitium and airspace, alveolar wall thickening, and appearance of proteinaceous debris in the alveolar space. These features worsened with age in *Sftpc*^−*/*−^ but not in *Sftpc*^*+/+*^ mice (Figure 1a). For the pathological evaluation of lung injury score (LIS), consistent with the American Thoracic Society (ATS) criteria for lung injury^40,41^, lung fields were randomly selected using a standardized digital method recently established^42^. LIS was significantly higher (worse) in *Sftpc*^−*/*−^ mice compared to age-matched *Sftpc*^*+/+*^ mice, where no changes over age were noted (Figure 1b; **Supplementary Figure 1a and b)**. Alveolar septa count (number of interalveolar septa), volume density of alveolar septa (an estimation of alveolar airspace), mean trans-sectional wall length (a measure of alveolar septal thickness), airspace surface area density (estimation of lung volume), but not mean linear intercept (estimation of volume to surface ratio of the airspace), were all significantly altered in *Sftpc*^−*/*−^mice with age (Figure 1c) and overall different from those of the corresponding *Sftpc*^*+/+*^ mice (Figures 1c and **bSupplementary Figure 1c)**, supporting the presence of a fibrotic remodeling process in *Sftpc*^−*/*−^ mice consistent with prior data^39,43^. The presence of interstitial thickening was further supported by elevated deposition of collagen (col) I and col IV (Figure 2a and **Supplementary Figure 2a**), and a significant age-dependent increased hydroxyproline [(2S,4R)-4-hydroxyproline, Hyp] content in *Sftpc*^−*/*−^ compared to *Sftpc*^*+/+*^ lungs (Figure 2b and **Supplementary Figure 2b)**. KO mice, lacking *Sftpc* and the related protein SPC, (Figure 2c-d), did not show a difference in the mRNA expression of either other surfactant genes (*Sftpa, Stfpb, Sftpd*) (Figure 2d) or other ATII cell markers, *Lysosomal Associated Membrane Protein 3* (*Lamp3)* and *Abca3* (Figure 2c and 2e). Taken together, our data indicate that lack of SPC results in morphological and stereological changes similar to histological patterns observed in chILD and worsening with age, while other ATII markers and surfactant genes do not appear clearly affected.

### Increased susceptibility to bleomycin in Sftpc^−/−^ mice.

**Figure 3.**
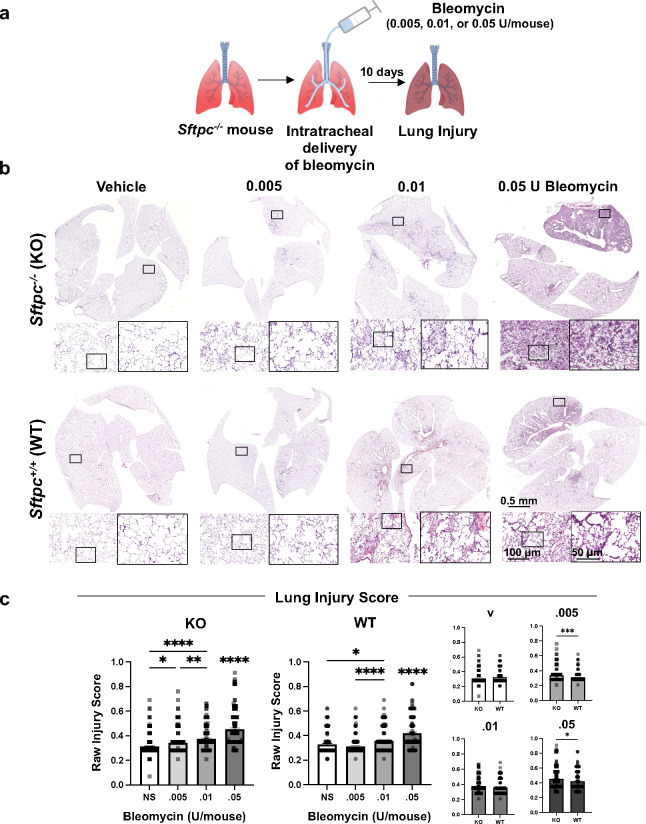
Susceptibility of *Sftpc*^*−/−*^ mice to bleomycin. **a.** Schematics of the injury model experiment with bleomycin. **b.** Representative histologic sections of whole lung comparing 4 months old *Sftpc*^*−/−*^ and *Sftpc*^*+/+*^ mice treated with increasing doses of bleomycin (0.005–0.05 U/mouse). Black squares indicate the area shown in the higher magnification. **c.** LIS evaluated on 1000×1000 μm lung histology fields (n=60 ROI/mouse, 3 mice/group). Data are reported as mean±SEM and analyzed by one-way ANOVA when the comparison is between different doses of bleomycin within the same strain and by Student t-test when KO is compered to WT (*, p=0.0304–0.0417; **, p=0.0094; ***, p=0.0007; ****, p<0.0001).

**Figure 4.**
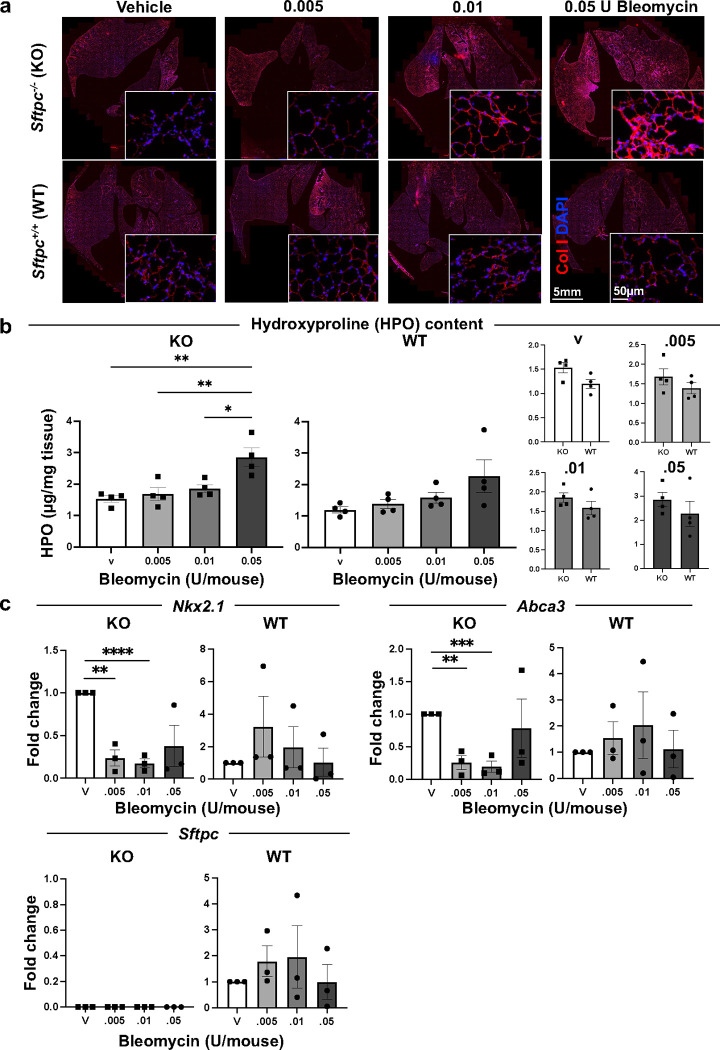
Lung collagen content and ATII cell markers analysis in *Sftpc*^*−/−*^ mice post-bleomycin treatment. **a.** Collagen I immunostaining of representative lung sections of 4 months old *Sftpc*^*−/−*^ and *Sftpc*^*+/+*^ mice treated with increasing doses of bleomycin (0.005–0.05 U/mouse). **b.** Hydroxyproline content quantification via colorimetric hydroxyproline. Data are reported as mean±SEM, n=4, and analyzed by one-way ANOVA when the comparison is between different doses of bleomycin within the same strain and by Student t-test when KO is compered to WT (*, p=0.0173; **, p=0.0024–0.0059). **c.** Expression by RT-qPCR of mRNA of alveolar epithelial markers. Data are reported as mean±SEM, n=3, and analyzed by Student t-test comparing each dose to vehicle within the same strain (**, p=0.0012–0.0023; ***, p=0.0008; ****, p<0.0001).

We next explored whether bleomycin could be used to partially ablate endogenous defective ATII cells in *Sftpc*^−*/*−^ mice. *Sftpc*^−*/*−^ mice on a Black Swiss background have normal lung structure at baseline^44^, but develop fibrosis after a low-dose (0.01U/mouse) of bleomycin compared to *Sftpc*^*+/+*^ mice^45^. In contrast, *Sftpc*^*−/−*^ mice on 129Sv background, described here, show interstitial pneumonitis at baseline (Figure 1 and ^39^) and develop extensive disruption of lung architecture and persistent lung inflammation after 0.05 U of bleomycin/mouse (a dose commonly used in *Sftpc*^*+/+*^ mice)^46^. We administered incremental doses of bleomycin (0.005, 0.01, and 0.05 U/mouse) i.t. to 4 mo *Sftpc*^*−/−*^ and *Sftpc*^*+/+*^ mice (Figure 3a). After 10 days, *Sftpc*^*−/−*^ mice showed increased alveolar wall thickening, interstitial neutrophils, hyaline membrane and proteinaceous debris lining alveolar walls at any dose of bleomycin while *Sftpc*^*+/+*^ mice showed initial histological evidence of lung injury at 0.01 U of bleomycin and clearly visible only at 0.05 (Figure 3b and **Supplementary Figure 3**). Compared to *Sftpc*^*+/+*^ mice, *Sftpc*^*−/−*^ mice had significantly higher LIS at any dose (Figure 3c and **Supplementary Figure 3**). Stereological analysis confirmed a bleomycin dose-dependent injury in *Sftpc*^*−/−*^ mice for septal count, septal density, and mean transactional wall length but not for mean linear intercept and airspace surface density **(Supplementary Figure 4)**. Nevertheless, we did notice that all the stereological values, except for septal counts, were significantly more altered in *Sftpc*^*−/−*^ compared to *Sftpc*^*+/+*^ for each bleomycin dose **(Supplementary Figure 4).** Elevated deposition of collagens (I and IV) and quantification of HPO were observed only at the highest dose of bleomycin in the *Sftpc*^*−/−*^ mice (Figure 4a-b and **Supplementary Figure 5a)**. RT-qPCR analysis showed downregulation of mRNAs encoding *Nkx2.1* as well as other ATII cell markers such as *Abca3* with increasing bleomycin dose In the *Sftpc*^*−/−*^ mice (Figure 4c and **Supplementary Figure 5b)**. We conclude that *Sftpc*^*−/−*^ mice are susceptible to bleomycin injury including its effect on ATII more than *Sftpc*^*+/+*^.

### Engraftment of syngeneic Sftpc^+/+^ ATII cells in bleomycin-conditioned Sftpc^−/−^ mice.

**Figure 5.**
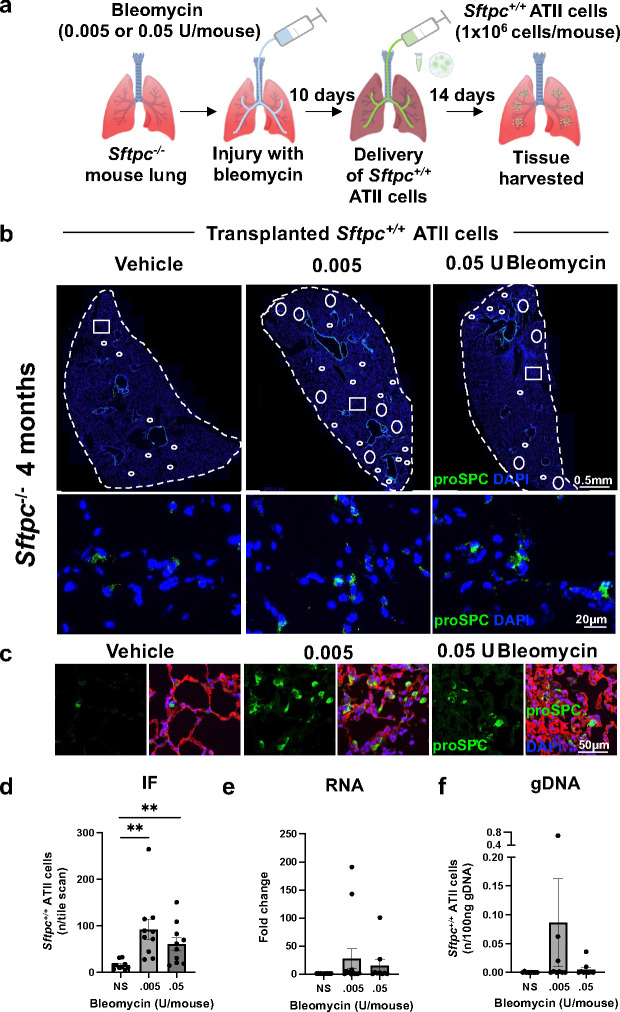
*Sftpc*^*+/+*^ ATII cells engraftment in bleomycin-conditioned *Sftpc*^*−/−*^ mice. **a.** Schematics of the *Sftpc*^*+/+*^ ATII cells transplant in *Sftpc*^*−/−*^ mice post-bleomycin conditioning. **b.** Immunostaining of lung sections for proSPC marker (transplanted and engrafted *Sftpc*^*+/+*^ ATII cells only) 14 days post-transplant in mice conditioned with bleomycin (0.005–0.05 U/mouse) or vehicle. Dotted lines highlight the areas of the analyzed lobes, circles show areas where the transplanted cells were found, squares indicated the areas shown in the higher magnification. **c.** Confocal images of alveolar regions showing the punctuated intracellular pattern of proSPC immunostaining within the transplanted ATII cells surrounded by ATI cells, identified by RAGE marker. **d.** ProSPC immunostaining quantification by manual count of proSPC^+^ cells in 20 ROIs/mouse, n=7–10. Data are reported as mean±SEM and analyzed by Student t-test comparing each bleomycin dose to vehicle (**, p=0.0025–0.0054). **e-f.** Quantification of transplanted *Sftpc*^*+/+*^ ATII cells by RT-qPCR *Sftpc* amplification in mRNA **(e)** and gDNA **(f)** extracted from lung homogenates. Data are reported as mean±SEM, n=7–10 and analyzed by Student t-test comparing each bleomycin dose to vehicle.

We next investigated engraftment of ATII cells in bleomycin-treated *Sftpc*^*−/−*^ mice. Primary ATII cells were isolated from *Sftpc*^*+/+*^ lungs, purified, and verified for epithelial markers and viability by fluorescence-activated cell sorting (FACS) (95–98% EPCAM^+^, CD45^−^) and immunostaining for pro-SPC **(Supplementary Figure 6a)**. 4, 8, and 12 mo *Sftpc*^*−/−*^ mice were conditioned with low-dose (0.005U/mouse) or high-dose (0.05U/mouse) bleomycin and 10 days later received 1×10^6^ *Sftpc*^*+/+*^ ATII cells i.t. (Figure 5a). In preliminary experiments, 1×10^6^ cells guaranteed consistent engraftment, 5×10^5^ cells resulted in fewer engrafted cells, while 2×10^6^ cells caused clumps of cells in the airways or inconsistent engraftment (data not shown). 14 days post-transplantation, lungs were harvested and analyzed for cell engraftment (Figure 5a). By counting pro-SPC+ cells from immunofluorescent (IF) staining of representative lung sections, we noticed higher engraftment of *Sftpc*^*+/+*^ ATII cells in with low-dose bleomycin when compared to vehicle or to the high-dose (Figure 5b-d). Pro-SPC counterstained with RAGE (ATI cell marker) showed ATII cells in proximity of ATI cells in their physiologic and anatomic context (Figure 5c).

Supporting the pro-SPC immunostaining, we found increased expression of *Sftpc* mRNA in mice that received low- compared to high-dose bleomycin (Figure 5e). To further estimate the percentage of transplanted cells, genomic DNA (gDNA) was extracted from lung sections derived from transplanted mice and subject to RT-qPCR analysis for *Sftpc* using a methodology previously applied technique for other transplantation models^47^. Using a standard curve of varying amounts of *Sftpc*^*+/+*^ cells alone **(Supplementary Figure 6b-d)** we estimated the engraftment of *Sftpc*^*+/+*^ cells per mouse. Again, we detected the higher number of transplanted *Sftpc*^*+/+*^ cells with low-dose bleomycin (Figure 5f). When we compare the engraftment efficient at different ages, we found that younger mice (4 and 8 mo) were engrafted more efficiently than older mice (12 mo) **(Supplementary Figure 7)**.

Overall, low-dose bleomycin, while causing a minimal lung injury, is permissive for robust *Sftpc*^*+/+*^ ATII cell engraftment especially in younger *Sftpc*^*−/−*^ mice.

### Long-term engraftment and functionality of Sftpc^+/+^ATII cells.

**Figure 6.**
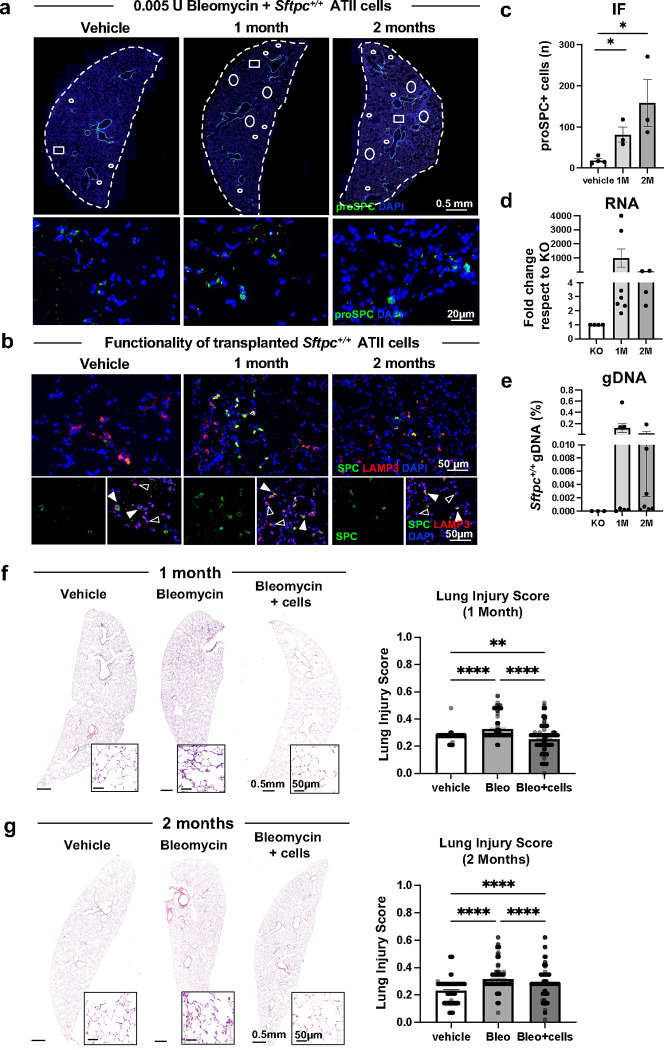
Long term viability and functionality of transplanted *Sftpc*^*+/+*^ ATII in bleomycin-conditioned *Sftpc*^*−/−*^ mice. **a.** Immunostaining of lung sections for proSPC marker (transplanted and engrafted *Sftpc*^*+/+*^ ATII cells only) 1 and 2 months post-transplant in mice conditioned with bleomycin (0.005U/mouse) or vehicle. Dotted lines highlight the areas of the analyzed lobes, circles show areas where the transplanted cells were found, squares indicated the areas shown in the higher magnification. **b.** Confocal images of mature SPC counterstained with Lamp3 showing engrafted *Sftpc*^*+/+*^ ATII cells (Lamp3^+^/proSPC^+^) are functional and capable of processing mature SPC in *Sftpc*^*−/−*^ mice lung. Representative transplanted *Sftpc*^*+/+*^ ATII cells are indicated with full arrows, while representative endogenous *Sftpc*^*−/−*^ ATII cells are indicated with empty arrows. **c.** ProSPC immunostaining quantification by manual count of proSPC^+^ cells in the whole lung tilescan, n=3–4. Data are reported as mean±SEM and analyzed by Student t-test comparing each time point to vehicle (*, p=0.0121–0.0332). **d-e.** Quantification of transplanted *Sftpc*^*+/+*^ ATII cells by RT-qPCR *Sftpc* amplification in mRNA **(d)** and gDNA **(e)** extracted from lung homogenates. Data are reported as mean±SEM, n=3–7 and analyzed by Student t-test comparing each long-term timepoint to mock-treated group (KO). **f-g.** Representative histologic sections of whole lung sections and LIS at 1 **(f)** and 2 **(g)** months post *Sftpc*^*+/+*^ ATII cells transplant and conditioned control with mock cells delivery. LIS is evaluated on 1000 × 1000 μm lung histology fields (n=60 ROI/mouse, 3–7 mice/group). Data are reported as mean±SEM and analyzed by one-way ANOVA (***, p=0.0008; ****, p<0.0001).

Next, we asked if primary *Sftpc*^*+/+*^ ATII cells could promote repair. 4 mo *Sftpc*^*+/+*^ mice were conditioned with low-dose bleomycin and 1×10^6^ *Sftpc*^*+/+*^ ATII cells were delivered i.t. 10 days later, lungs were harvested and analyzed at 4 and 8 weeks post-transplantation. By immunostaining, we could detect a significant amount of pro-SPC+ ATII cells in the alveolar region of all the *Sftpc*^*−/−*^ mice conditioned with bleomycin compared to those conditioned with vehicle only (Figure 6a). In transplanted mice, we could detect patches of LAMP3+ SPC+ ATII cells, indicating that transplanted *Sftpc*^*+/+*^ ATII cells were not only viable, but also capable to fully processing pro-SPC in its mature product, SPC (Figure 6b), as an indirect sign of their physiological recovery into the recipient lungs. By IF and mRNA and gDNA analysis for *Sftpc*, we confirmed the persistence of *Sftpc*^*+/+*^ cells up to 8 weeks (Figure 6c-e). Finally, we asked whether transplanted ATII cells could promote repair of lungs injured by bleomycin. Histological analysis of mice lungs at 4 weeks post-transplantation suggested improved LIS when compared not only to mice conditioned with bleomycin but also to controls (vehicle and mock cell delivery) (Figure 6f). At 8 weeks, transplanted mice had an improved LIS compared to mice conditioned with bleomycin (Figure 6g). Taken together, these data indicate that transplanted ATII cells can attenuate lung injury induced by bleomycin and early they can also halt the disease progression.

## Discussion

No prior examples of cell therapy have used an ATII cell disease model as in this case. Using a chILD-like disease model, the 129/Sv *Sftpc*^*−/−*^ model, we found that *Sftpc*^*+/+*^ ATII cells can engraft after pre-conditioning with low-dose bleomycin, especially early in the course of the disease, and engrafted *Sftpc*^*+/+*^ ATII cells are capable of repairing lung injury in those mice.

The initial study of Glasser and colleagues reported the generation of *Sftpc* knock-out mice on a Swiss black background^44^. Those mice were viable at birth and developed normally with only mild alterations in lung mechanics and decreased stability of surfactant when analyzed *in vitro*. In contrast, the same mutation on a 129/Sv background, used for the current studies, induced ATII hyperplasia with LB-like and lipid inclusions, increased neutrophils, alveolar macrophages with intracellular surfactant-like material, accumulation of a-smooth muscle actin (a-SMA) and are, therefore, a better model of the human disease^39^. Importantly, these mice are normal at birth, and progressively exhibit signs of interstitial fibrosis and cellular inflammation that progress with age with extensive remodeling, airspace loss and patchy fibrosis, and show low fertility rate^39^. Other animal models carrying *Sftpc* mutations exist, including a mouse expressing most common *SFTPC* mutation found in patients, *SFTPC*^*I73T*^.^17^ The *Sftpc*^*I73T*^ mouse model^,^ results in embryonic lethality unless inducibly expressed in the post-natal period^17,48^. After tamoxifen induction, these mice develop severe polycellular alveolitis with increased mortality between 7 and 14 days. This is in direct contrast with children affected by child-*SFTPC*^*I73T*^, in whom symptoms appear variably after birth and not typically associated with postnatal mortality^49,50^. Other variants of *SFTPC* in humans include L188Q, Δexon4, and C121G, all localized to the pro-SPC COOH-terminal (BRICHOS) domain^18,51^. They cause protein misfolding, aggregation, ER stress and apoptosis^18,51^. Mice expressing Δexon4 or the C121G *SFTPC* mutant show ATII cell death, fibrotic remodeling, and neonatal lethality^52,53^. When C121G mutant is expressed in an inducible manner, mice show a pattern of chronic fibrosis^40^. The mouse expressing L188Q and a recent knock-in model expressing this mutation, do not develop lung fibrosis unless conditioned with bleomycin^54,55^. In contrast to these inducible models, the model used in the current study showed a consistent phenotype across the experiments (Figure 1 and 2). Our model is therefore currently the best approximation of a pre-clinical model for cell therapy of chILD.

Bleomycin remains the most frequently used agent to induce pulmonary fibrosis in animal models. The fibrotic response of *Sftpc*^−/−^ mice to bleomycin has already investigated in two previous studies^45,46^. Here instead, low-dose bleomycin was used as a conditioning strategy to partially depleting ATII cells prior to cell transplant. In fact, 0.005U/kg of bleomycin (one-tenth of the dose normally used in wild-type mice) was sufficient to partially affect ATII cells without severely further damaging lungs (Figures 3 and 4) and allow exogenous ATII cell engraftment (Figure 5 and 6). We also showed that without proper conditioning of the lung epithelium, even when defective, as in *Sftpc*^*−/−*^ mice, cell engraftment is minimal, if not completely absent. While bleomycin fits the purpose of animal studies, we are aware that alternative conditioning methods need further investigation to be applicable to humans.

When considering cell therapy, the first question is the choice of engrafting cells. Recent studies have shown that murine primary, embryonic or pluripotent stem-cell derived cells can be transplanted in recipient murine lungs post injury and cells persisted up to 4–6 months^33,56–64^. Human primary and pluripotent stem-cell derived cells have been transplanted into mice airways, and in one case, into distal lungs^58,60,62,65^. In all these previous studies, wild-type or immunodeficient mice were used as recipient; no previous models of cellular therapy have utilized an ATII cell disease models as in this study (see **Supplemental Table 1**).

Here, we used highly purified *Sftpc*^*+/+*^ ATII cells as a proof-of-concept study and a disease model as recipient. Transplanted primary ATII cells have previously been shown to promote a certain degree of lung recovery post-injury in wild-type mice^33,63^. The ATII cells transplanted in our study present several advantages. They are isolated from *Sftpc*^*+/+*^ mice lungs and they express SPC. Therefore, when transplanted in *Sftpc*^*−/−*^ mice, those ATII cells can potentially correct the phenotype of the host, reintroducing SPC. They are easily identifiable in the *Sftpc*^*−/−*^ by IF for pro-SPC and by RT-qPCR for *Sftpc* facilitating any engraftment analysis without requiring external manipulations for identification (e.g. lentiviral fluorescent labeling). Finally, since they are syngeneic, the host does not require any immunosuppression to allow engraftment. Multipotent Sox9^+^ murine lung progenitors have been isolated from fetal lungs and showed to engraft in immunocompromised mice after injury and differentiate in airway and alveolar cells^64^. Recently, another group has been able to derive and expand *in vitro* Nkx2–1^+^/Sox9^+^ lung epithelial progenitors from murine embryonic stem cells (ESCs), called ESC-derived tip-like cells, and showed differentiation into ATII-like and ATI-like cells when transplanted into the lungs of syngeneic immunocompetent recipients^61^. While these results are promising, they have not yet been applied to a disease model nor evaluated for therapeutic effect on disease progression.

A second question is defining the number of defective cells to be replaced to demonstrate a therapeutic benefit for patients. Replacing all ATII cells may be neither safe nor needed. For example, in *Sftpb*^*−/−*^ mouse models, symptoms appear only when SPB levels fall below 20–25% of wild-type levels^66^. Our data support the idea that partial replacement of ATII cells is sufficient to promote lung repair, as we transplanted up to 1×10^6^ ATII cells, one tenth of total ATII cell number in a mouse lung^67^, which was sufficient to attenuate lung injury secondary to bleomycin (Figure 6).

The third question is timing of intervention. All the current therapies of chILD aim to attenuate the downstream effects of the disease but do not target the main cell involved in its pathogenesis, the ATII cell^18,68^. In replacing or correcting defective ATII cells, timing is critical since it should ideally occur prior to irreversible chronic changes of the lung. In this study, we transplanted animals at 3 different ages (4, 8, and 12 mo) and used bleomycin as pre-conditioning strategy. We noticed that at 4 and 8 mo, low-dose of bleomycin allowed better engraftment among all the conditions tested **(Supplementary Figure 7)**. In 12 mo mice, instead, cell engraftment was always lower for any bleomycin dose and variable between experiments respect to younger mice. This could be explained by the fact that the natural progression of lung disease of the *Sftpc*^*−/−*^ mice aggravated by bleomycin creates a hostile milieu for cell attachment and engraftment. Our data suggested that cell therapy for progressive lung diseases such as chILD should occur early in the disease course.

We observed long-term engraftment and attenuation or repair of bleomycin-induced injury (Figure 6f-g). Our data support those of other groups on mouse-to-mouse cell transplants using bleomycin^33,57,58^. Louie and colleagues showed that Rag1 knock-out mice conditioned with bleomycin and transplanted with organoid cells derived from SCA1-negative ATII cells had lower Ashcroft injury scores compared to those conditioned only with bleomycin^58^. Mice conditioned with bleomycin and transplanted with club-like lineage negative epithelial progenitors high in H2-K1 expression showed improved oxygen saturation at 2 weeks post-transplant^57^. In a similar study, mice transplanted with primary ATII cells post-bleomycin improved their oxygen saturation and their arterial partial pressure of oxygen at 2 weeks post-transplant^33^. Regardless of the mechanisms and signals involved, the current and other studies^33,57,58^ showed that transplanted ATII or ATII-like cells could promote repair or functional recovery of the lung. We observed that one month after transplantation, mice receiving ATII cell transplant exhibited a marked improvement in LIS compared to both untreated mice (experiencing the natural progression of the disease) and mice treated with bleomycin (experiencing injury). However, by the two-month mark, the disease improvement in ATII cell-transplanted mice was primarily noticeable in comparison to the mice injured with bleomycin (Figure 6). This might suggest that the disease naturally progresses faster than the regenerative capacity of transplanted cells. Future studies should explore whether transplanting subpopulations of ATII cells with high progenitor-capabilities (such as Wnt-responsive Axin2-expressing subset of ATII cell)^69,70^ or murine ESC-derived tip-like cells^61^ could yield prolonged and persistent benefits in chILD-like disease models. Nevertheless, a single cell transplant might not be adequate to entirely and permanently halt the progression of the disease and sequential cellular transplants might be necessary to effectively manage the disease over time.

Our study has several limitations. While bleomycin is commonly used as pre-conditioning strategy in animal models^33,57,58,61^, this conditioning might be challenging to apply in patients. Another limitation is that it is challenging to accurately determine the total number of engrafted *Sftpc*^*+/+*^ ATII cells in the entire mouse lung. While *Sftpc*^*+/+*^ ATII cells could be identified by pro-SPC immunostaining, the lack of lineage tracing of our transplanted cells did not allow us to follow the fate of these cells or determine if they differentiated into other lung cell types, including ATI cells. This could lead to underestimate the number of cells derived from the initially engrafted *Sftpc*^*+/+*^ ATII cells. Nonetheless, our study demonstrates the successful engraftment, persistence of ATII cells, and their repairing capacity in a chILD-like disease model using low-dose bleomycin as conditioning strategy. Ultimately, *Sftpc*^*−/−*^ mice with a minimal conditional strategy, such bleomycin, could represent a preclinical, translational platform to assess cell therapy in chILD. In conclusion, our study lays the foundation for cell therapy in chILD, offering an alternative approach to lung transplantation.

## Methods

### Animals.

129S2/SvPasOrlRj (129/Sv) *Sftpc*^*−/−*^ mice, generated by gene inactivation as previously described^39^, were generously donated by Dr. Whitsett, Cincinnati Children’s Hospital. 129/Sv *Sftpc*^*+/+*^ (wild-type) mice used as control group were purchased by Taconic Biosciences (NY, USA). In all the experiments, 4-, 8-, or 12-month-old mice, both female and male, were used. All animal work was approved by the Columbia University Institutional Animal Care and Use Committee and complied with the National Research Council *Guide for the Care and Use of Laboratory Animals*. Mice were humanely euthanized via inhalation of 5% isoflurane, followed by a second method of euthanasia: cervical dislocation followed by median sternotomy. Lungs were perfused via right ventricle with PBS (Phosphate Buffer Saline), and following perfusion, an incision was made, and the trachea was cannulated using a 20G catheter. The catheter was secured in position via a suture wire, and the lungs were filled by intratracheal instillation of 80% OCT (optimal cutting temperature compound, Sakura Finetek)/20% PBS, removed surgically from the animal, frozen, sectioned according to pre-determined map^42^ and stored at −80°C.

### Delivery of Bleomycin and Induction of Lung Injury.

15 Units (U) of bleomycin powder (Meitheal Pharmaceuticals Inc.) was resuspended in 5 mL of sterile normal saline (NS) solution and stored sterile at 4°C. For any use, only the necessary amount was taken from the vial. 4–12 months-old 129/Sv *Sftpc*^*+/+*^ and *Sftpc*^*−/−*^ 129/Sv mice were weighed and anesthetized with an intraperitoneal injection of Ketamine (80–95 mg/kg) and Xylazine (5–10 mg/kg). Intratracheal intubation was performed using a 20G cannula modified in length for murine lungs. Bleomycin was administered at 0.005, 0.01, or 0.05 U/mouse in a total volume of 40μL in sterile NS. Control mice received 40μL of vehicle (NS). Following i.t. administration, mice were allowed to recover from anesthesia and returned to their cages until euthanasia. Endpoint analysis was performed at 10 days from bleomycin injection. Mice were humanely euthanized with isoflurane and lung tissues were harvested, frozen in OCT, and stored at −80°C. Tissues were processed for Hematoxylin and Eosin (H&E), immunofluorescent staining, or gDNA/mRNA extraction.

### Stereological Analysis and Lung Injury Score.

H&E sections were prepared by the Herbert Irving Cancer Center Molecular Pathology Core. Sections were scanned on a Leica AT2 slide scanner at 40X resolution (.25 microns/pixel). 1000 × 1000 μm sections were randomly generated using a ImageJ macro as previously described^42^ and then were analyzed for morphometric and stereological features, and LIS. For each analysis, 60–120 random sections were analyzed for each condition. Vvsep, Vsair, Lm, and Lmw were calculated according to the methods previously described^71,72^. Septal counts were performed manually by two independent researchers blind to the treatment group using the grid system previously described by our group^73^. For LIS, these same sections were analyzed by a pathologist blinded to the treatment group according to ATS guidelines^40,41^ to determine lung injury^42^.

### Hydroxyproline Assay.

Hydryoxproline content was determined for each sample using Millipore Sigma Hydroxyproline Assay kit (MAK008). 25–30 mg of tissue was sectioned from tissue-blocks cryopreserved in OCT. Sections were washed in PBS to remove residual OCT, weighed, dried, hydrolyzed, and ran according to manufacturer’s protocol.

### Isolation of ATII Cells.

Healthy 129/Sv *Sftpc*^*+/+*^ mice at 2–3 months of age were humanely euthanized for isolation of *Sftpc*^*+/+*^ ATII cells. Lungs were perfused with PBS via the right ventricle of the heart and bronchioalveolar lavage was performed with PBS prior to inflate lungs *in situ* with Dispase (~0.9 ml, 50U/ml, Corning #354235) via tracheal cannulation. Lungs were tied with suture wire and incubated in an additional 1 mL of Dispase at room temperature (RT) for 20 min. Following incubation, pulmonary lobes were separated from the trachea and bronchial tree and chopped mechanically with a variety of surgical scissors. After dissection and mechanical separation, the tissue was incubated at 37°C in MEM + DNase I with frequent agitation to complete digestion for 10 min. The mixed cell digest was filtered through 100 μm and 40 μm cup filters, and fibroblasts were removed from the suspension by three successive adherence steps on tissue-culture treated plastic. Alveolar epithelial type II cells were then purified from resident macrophages, lymphocytes, and blood cells through two passes of cell enrichment and isolation kits (Dynabeads DC Cell Enrichment Kit #114.29D; Dynal Mouse T Cell Negative Isolation Kit #114.13D). Kits were used as described in manufacturer protocol. The purity of the resulting ATII population was verified both via immunostaining (pro-SPC) after plating overnight the cells in Matrigel (1:30 mixed in media), and by selecting CD45− EpCAM+ population (CD45 BV421, Biolegend #103133; EpCAM PE/Cy7, Biolegend #118215) using a Sony MA900 cell sorter (San Jose, CA).

### Bleomycin Conditioning and ATII Cell Engraftment.

4-, 8-, or 12-months old *Sftpc*^*−/−*^ 129/Sv mice were anesthetized and treated with 0.005–0.05 U of bleomycin, or vehicle in 40μL volume via i.t delivery as previously described in this paper. Each age group contained 3 animals for each treatment condition. After 10 days, mice received 1×10^6^ freshly isolated *Sftpc*^*+/+*^ ATII cells delivered i.t. and were harvested at 2–16 weeks post cell delivery. Lung tissues were inflated with 80% OCT/20% PBS and sectioned into upper, middle, and lower lobes according to a predetermined lung map that covers both lungs from upper to lower regions as previously described^42^.

### Immunofluorescent Staining.

Lung sections were thawed at RT for 5–10 minutes, fixed with 4% paraformaldehyde for 10 minutes at room temperature (RT) and washed with PBS for 5 minutes. The sections were permeabilized with 0.25% Triton X-100/PBS for 20 minutes followed by blocking in 10% donkey serum diluted in PBS for 1 hour. Primary antibodies **(Supplementary Table 2)** were diluted in 5% donkey serum in PBS and incubated at 4°C overnight. The following day, sections were washed with PBS + 0.025% Triton-X for three times, 5 minutes each followed by secondary antibody **(Supplementary Table 3)** incubation for 1 hour at RT. Following secondary incubation, sections were washed three times for 10 minutes with PBS + 0.025% Triton-X100 and finally mounted with DAPI contained fluorescent mounting medium. The following antibodies were used: proSPC (Seven Hills, #WRAB-9337), mature SPC (Seven Hills, #WRAB76694), RAGE/AGER (R&D, #AF1145), Collagen I (abcam, #ab34710), Collagen IV (abcam, #ab6586), vimentin (abcam, #ab92547), LAMP3 (Synaptic Systems, #391005), and anti-TTFI (Seven Hills, WRAB-1231), Donkey a Rabbit IgG 488 (Invitrogen, #A21206), Donkey α Rabbit IgG 555 (Invitrogen, #A31572), Donkey α Rabbit IgG 647 (Invitrogen, #A31573), Donkey α Goat IgG 568 (Invitrogen, #A11057), and Goat α Guinea Pig IgG 555 (Invitrogen, #A21435) with the dilutions according to the tables **(Supplementary Tables 2** and **3)**. For proSPC and mature SPC staining in the transplant experiments analysis, TrueVIEW^®^ Autofluorescence Quenching Kit (Vector, SP-8400–15) was applied and counterstained with DAPI prior to imaging. Samples were imaged using motorized DMi8 (Leica Microsystems, Buffalo Grove, IL) inverted microscopes. Confocal images were taken on a Leica Stellaris 8. For NKX2.1 stainings, 20 random 500 × 500 μm regions of interest were generated for each sample and positive cells were counted over total number of cells.

### RT-qPCR.

200 μm thick slices of lung tissue cryopreserved in OCT were sectioned and washed with PBS to remove residual OCT. For mRNA extraction, tissue was digest and homogenized in Trizol reagent according to manufacturer’s protocol (Invitrogen^™^, #15596026). For RT-qPCR of mRNA expression, mRNA was extracted using Zymogen Direct-zol RNA Microprep kit (#R2062), and cDNA was generated from RNA extracts using the Multiscribe High-Capacity cDNA kit (ThermoFisher, #4368814). For the analysis, 20 ng of cDNA was analyzed per well. For genomic DNA extraction, tissue was lysed, and gDNA was extracted using Zymogen Quick-DNA Microprep Kit (#D3021) according to manufacturer’s protocol. For the analysis, 100 ng gDNA was analyzed per well. DD Comparative qPCR was run on all samples using SYBR green reagents (ThermoFisher, #A25743) and using gene-specific PCR primers from Integrated DNA Technologies **(Supplementary Table 4)**.

### Data availability.

The data that support the findings of this study are available on request from the corresponding author, NVD.

### Statistical methods.

Statistical methods applied for each experiment are outlined in the figure legends. Both male and female mice were used with no clear difference in response to bleomycin or transplantation. Unpaired, two-tailed Student’s t tests were used when comparing two groups, while ANOVA and Tukey’s test were used when comparing multiple groups. For each data set, mean±SEM were calculated and presented in the legend section of each figure. The differences between the groups were considered statistically significant for p ≤ 0.05. If not indicated, the difference between groups is not statistically significant.

### Study approval.

Mice were housed in a pathogen-free barrier facility at Columbia University Vagelos College of Physicians and Surgeons. All animal procedures were performed under protocol (#AC-AABP0553) approved by the Institutional Animal Care and Use Committee of Columbia University Vagelos College of Physicians and Surgeons.
